# Non-invasive prenatal test to screen common trisomies in twin pregnancies

**DOI:** 10.1186/s13039-020-0475-8

**Published:** 2020-02-05

**Authors:** Mahtab Motevasselian, Soraya Saleh Gargari, Sarang Younesi, Parichehr Pooransari, Pourandokht Saadati, Masoomeh Mirzamoradi, Shahram Savad, Mohammad Mahdi Taheri Amin, Mohammad-Hossein Modarresi, Maryam Afrakhteh, Soudeh Ghafouri-Fard

**Affiliations:** 1 Men’s Health and Reproductive Health Research Center, Shahid Beheshti University of Medical Sciences, Tehran, Iran; 2 Prenatal Screening Department of Nilou Laboratory, Tehran, Iran; 3 Department of Medical Genetics, School of Medicine, Tehran University of Medical Sciences, Tehran, Iran; 4 Department of Medical Genetics, Shahid Beheshti University of Medical Sciences, Tehran, Iran

**Keywords:** NIPT, Non-invasive prenatal testing, Cell free DNA, Trisomy 21, Trisomy, 18, Trisomy 13, Twin pregnancy

## Abstract

**Objectives:**

Recent years have witnessed a shift from invasive methods of prenatal screening to non-invasive strategies. Accordingly, non-invasive prenatal testing (NIPT) using cell-free fetal DNA in maternal plasma has gained a considerable deal of interest from both geneticists and obstetricians. Efficacy of this method in identification of common aneuploidies has been extensively assessed in singleton pregnancies. However, a limited number of studies have addressed the twin pregnancies. In this context, the present study is aimed at identification of the efficacy of NIPT in twin pregnancies.

**Methods:**

NIPT was performed on twin pregnancies to screen trisomies 13, 18 and 21. Pregnant women referring to Nilou Clinical Laboratory between March 2016 and December 2018 were included in this research.

**Results:**

In the current study, a total 356 twin pregnancies were screened in search for trisomies 13, 18 and 21. 6 cases exhibited positive NIPT results in which the presence of trisomies 13, 18 and 21 was confirmed by fetal karyotype in 1, 2 and 2 cases, respectively. One twin pregnancy showed normal karyotype. The combined false-positive rate for these trisomies was 0.28%. No false negative case was observed. The combined sensitivity and specificity of NIPT in twin pregnancies were 100 and 99.7%, respectively.

**Conclusion:**

The results of the current study verify the feasibility, sensitivity and specificity of NIPT in twin pregnancies.

## Introduction

Non-invasive prenatal testing (NIPT) using cell-free fetal DNA in maternal plasma has been successfully employed for aneuploidy screening in clinical settings [1]. Several studies have assessed the accuracy of this method in singleton pregnancies. A meta-analysis of 35 relevant studies has indicated that NIPT is able to detect more than 99% of trisomy 21 cases, 98% of trisomy 18 cases and 99% of trisomy 13 cases in singleton pregnancies at a combined false positive rate (FPR) of 0.13% [2]. Such high detection rates firmly supported the application of this test in clinical settings for singleton pregnancies. However, reports on twin pregnancies are scarce. Tan et al. assessed sensitivity and specificity of NIPT in twin pregnancies resulted from assisted reproductive technology (ART). Their findings revealed a failure rate of 0.9%, (5/565), 100% positive predictive value and no false negative result. Accordingly, they proposed NIPT as an appropriate strategy for prenatal screening of ART twin pregnancies [3]. Villela et al. employed NIPT for sex determination in twin pregnancies. Their analytical method exhibited 100% sensitivity and specificity when both twins were female, while the presence of a male co-twin declined the sensitivity and specificity to 98 and 95%, respectively [4]. More recently, Yang et al. successfully applied NIPT for screening more than 400 twin pregnancies including both double chorionic dichorionic diamniotic (DCDA) and monochorionic diamniotic (MCDA). The combined FPR was 0% for trisomies 21 and 18 with no false negative. Altogether, the combined sensitivity and specificity values were 100 and 99.53%, respectively. Despite the high performance of NIPT in detection of trisomy 21 in twin pregnancies, they mentioned the necessity of high quantities of clinical samples to validate the applicability of NIPT for other aneuploidies rather than trisomies 21 and 18 in both singleton and twin pregnancies [5].

In this regard, the current investigation is aimed to evaluate the performance of NIPT in identification of trisomies 13, 18 and 21 in twin pregnancies using the data available from a single referral clinical laboratory in Tehran, Iran.

## Methods

### Samples collection

In this study, NIPT was conducted on 500 twin pregnancies to screen trisomies 13, 18 and 21. Pregnant women referring to Nilou Clinical Laboratory between March 2016 and December 2018 were included in the research. Pretest counseling was provided for all pregnant women. Written informed consent was also obtained from all the participants. Moreover, the study was approved by the ethical committee of Shahid Beheshti University of Medical Sciences.

### NIPT

After sampling 5 mL of peripheral blood from pregnant women into the EDTA tubes, the plasma was separated according to the double-centrifugation method. In a typical procedure, blood samples were centrifuged twice at 1600 g for 10 min at 4 °C and 16,000 g for 10 min, respectively to eliminate the remaining cells [6]. The QIAamp kit (QIAGEN, Hilden, Germany) was used to extract the circulating DNA in a vacuum manifold. End-repair enzymes were employed to fill the sticky-ended DNA fragments and produce blunt-ended fragments. 5′-end phosphorylation was carried out to facilitate the oligonucleotide adapters binding during the library preparation. Subsequent incubation could lead to the denaturation of end-repair enzymes. Next, sequencing adaptors with unique barcodes, DNA ligase and DNA polymerase were added to each sample. IONA® Library Preparation Kit was also applied at this stage. After removing the unused adaptors, paramagnetic beads were employed to seize the DNA dissolution. Subsequent amplification was performed with a high-fidelity DNA polymerase and primers that bound to the adaptor sequences. The resultant libraries were quantified based on the method described previously [7]. Ion Torrent (Life Technology) genome analyzer was used for massively parallel sequencing.

### Bioinformatics analysis

Bioinformatic analysis was performed based on the method described by Crea et al. using IONA software [7]. In brief, the following steps were conducted: retrieval of multiplexed sequence reads, barcode classification, initial quality filtering to eliminate ultra short reads, trimming step, mapping to the human reference genome, filtering of aligned sequences and adjustment of sequencing coverage bias based on GC content. At last, likelihood ratios were calculated using the fragment count statistics and models that combined distributions of estimated values under both trisomy-affected and unaffected assumptions for trisomy 13, 18, and 21 tests [7].

### Fetal karyotype

Amniocentesis was performed on the screen-positive cases. Routine karyotyping was also carried out on the metaphase chromosome. Approximately, 300 to 400 bands were detectable. The sensitivity and specificity of NIPT were calculated using this method.

## Results

NIPT was conducted on 500 twin pregnancies (424 DCDA, 69 MCDA and 7 monochorionic-monoamnionic (MCMA) twin pregnancies). Indications for NIPT were as follows: maternal request (*n* = 382), positive results of the first trimester screening (*n* = 70), intermediate risk in the first trimester screening (*n* = 31), positive results of the second trimester screening (*n* = 8), intermediate risk in the second trimester screening (*n* = 4), positive results of sequential tests (*n* = 2), the history of Down syndrome (*n* = 1) and cerebral palsy (n = 1) among the first-degree relatives. Table 1 summarizes the demographic data of the enrolled participants.

**Table 1:** The available demographic data of enrolled pregnant women

Parameters		Values
Maternal age (mean ± SD)		34.5 ± 3.1
Weight (mean ± SD)		70.4 ± 5.1
Height (mean ± SD)		159 ± 7.1
Body Mass Index (mean ± SD)		27.846 ± 1.3
Fetal fraction (mean)		10.5%
Gestational age at sampling (mean ± SD)		15w + 4d (±5d)
Gestational age at delivery^a^(mean ± SD)		35w + 5d (±4d)
Gestational diabetes (number (%))		102 (20.4%)
Hypothyroidism (number (%))		98 (19.6%)
Type of pregnancy (number (%))	Spontaneous	317 (63.4%)
	IVF-ICSI	173 (34.6%)
	IUI	10 (2%)

^a^Based on the data of 356 pregnancies

Subsequently, 144 pregnancies (28.8%) were excluded due to the several reasons: 94 patients (18.8%) did not come for follow-up, no karyotype could be achieved in 22 of twin pregnancies (4.4%), 7 cases (1.4%) led to intrauterine death of both fetuses among which two IVF pregnancies existed, 2 twin pregnancies (0.4%) were subjected to selective embryonic reduction, and 19 cases (3.8%) were terminated due to preterm labor (*n* = 11), premature rupture of membranes (n = 7) and severe preeclampsia (*n* = 1). Six out of 19 terminated pregnancies were IVF pregnancies. All the excluded cases had normal NIPT results. The specificity and sensitivity of NIPT were calculated based on the results obtained from the remaining 356 twin pregnancies. Tables 2 and 3 summarize the clinical details of 6 cases with fetal trisomies and the only case with false positive NIPT results, respectively. Four of the trisomic cases (cases 2–5) had different genetic outcomes in twins and in one case (case 1) both twins had trisomy 21. Based on the results, these twin pregnancies were DCDA. In one case, fetal fraction value was lower than the appropriate value for NIPT (fetal fraction = 4%); hence the NIPT results were low risk (Case 5). Anomaly scan reported the presence of a choroid plexus cyst in one fetus. Subsequent amniocentesis confirmed trisomy 18 in one fetus and normal karyotype in the other. There were 6 cases of positive NIPT results in which the presence of trisomies 13, 18 and 21 was confirmed by fetal karyotype in 1, 2 and 2 cases, respectively. We failed in the follow-up of one case (Case 6). One twin pregnancy had normal karyotype (Table 3). The combined false positive rate for these trisomies was 0.28%. Post-labor follow-up showed no other false negative case. The combined sensitivity and specificity of NIPT in twin pregnancies were 100 and 99.7% respectively.

**Table 2:** Clinical details of the 6 cases with fetal trisomies (FTS: first trimester screening)

	Results of screening tests	Maternal age (years)	Gestational age	Conception	Nuchal translucency	Fetal fraction (%)	Maternal weight (Kg)	NIPT Result	Karyotyping
Case 1	T21 (QUAD test): 1/723 & 1/89	35	16w + 2d	Spontaneous	1.04 mm & 2.2 mm	9	63	T21 High Risk	47,XX,+ 21, 47,XX,+ 21
Case 2	T21 (FTS): 1/2049 & 1/576	45	15w + 1d	Spontaneous	2.3 mm & 2.1 mm	8	61	T13 High Risk	47,XX,+ 13, 46,XX
Case 3	T21 (Sequential): 1/1423 & 1/611 T21 (FTS): 1/6300 & 1/2021	32	12w + 6d	IVF-ICSI	1.2 mm & 1.9 mm	5	73	T18 High Risk	47,XX,+ 18, 46,XX
Case 4	T21 (FTS): 1/78 & 1/125 T13 (FTS): 1/330 & 1/723	32	13w + 2d	Spontaneous	3.01 mm & 2.8 mm	7	65	T21 High Risk	47,XX,+ 21, 46,XX
Case 5	T21 (FTS): 1/1821 & 1/897 T18 (FTS): 1/898 & 1/1002	41	12w + 2d	IVF-ICSI	1.3 mm & 1.5 mm	4	77	Low risk	47,XX,+ 18, 46,XX
Case 6	T21 (FTS): 1/969 & 1/103	36	21w + 3d	Spontaneous	1.7 mm & 2.5 mm	10	52	T21 High Risk	Unknown

**Table 3:** Clinical details of false positive NIPT results

Results of screening tests	Maternal age (years)	Gestational age	Conception	Nuchal translucency	Fetal fraction (%)	Maternal weight (Kg)	NIPT Result	Karyotyping
T21 (FTS): 1/473 & 1/1523	38	13w + 5d	IVF-ICSI	1.9 mm & 1.6 mm	5	70	High Risk / T21 > 95%	Normal

## Discussion

A limited number of studies have addressed the feasibility and accuracy of NIPT in twin pregnancies. A recent assessment of the screening data from two populations presented a comprehensive meta-analysis of peer-reviewed papers on the clinical confirmation or application of NIPT for trisomies 21, 18 and 13 in twin pregnancy. The mentioned paper indicated the similar performance of NIPT for trisomy 21 detection for singleton and twin pregnancies. The method performed better than the first-trimester combined test or second-trimester biochemical test. However, based on the low frequency of trisomy 18 and 13 cases, the author stated that the predictive performance of NIPT can’t be reported with high accuracy [8]. Table 4 summarizes the results of studies reporting the efficacy of NIPT in twin pregnancies.

**Table 4:** Results of studies indicating the efficacy of NIPT in twin pregnancies. All the studies used massively parallel sequencing. (NA: not assessed; DC: dichorionic, MC: monochorionic)

Authors	Number of pregnancies	Chorionicity (DC/ MC)	Conception (spontaneous/ART)	BMI	Maternal age (years)	Gestational age at sampling	Fetal fraction (%)	Sensitivity for trisomy 21 (%)	False positive (number)
Gil et al. [9]	68	52/ 16	22/ 46	NA	37.2	10.6	7.4 (median of lower fetal fraction)	100	0
Huang et al. [10]	189	152/ 33	70/ 113	NA	31	19	NA	100	NA
Gromminger et al. [11]	38	15/ 5	23/ 15	NA	NA	14.2	14.8	100	0
Bevilacqua et al. [12]	515	301/ 67	243/ 272	NA	36.8	13.6	8.7 (median of lower fetal fraction)	91.6	0
Tan et al. [3]	565	544/ 18	0/ 565	NA	31	12	8.9	100	0
Sarno et al. [13]	438	373/ 65	192/ 246	23.5	37.3	11.7	8 (median of lower fetal fraction)	100	1
Fosler et al. [14]	487	NA	NA	NA	35.5	16.6	16.1	100	1
Du et al. [15]	92	53/ 39	52/ 40	NA	30.5	17.9	20.65	100	0
Le Conte et al. [16]	492	387/ 101	301/ 184	22.9	37	16.3	13.4	100	1
Yang et al. [5]	432	337/ 95	93/ 235	NA	NA	NA	NA	100	2
Present study	500 (complete follow-up for 356 cases)	424/ 76	317/ 183	27.846	34.5	15.6	10.5	100	1

In the current assessment on the performance of NIPT method in 354 twin pregnancies, no false negative result (except for the case with a fetal fraction of 4%) was reported; there existed one false positive case which was an IVF-ICSI pregnancy. This point further highlights the complexity of the interpretation of NIPT results in pregnancies by assisted reproductive techniques. There was no significant difference in detected trisomies between spontaneous and IVF-ICSI pregnancies. Based on the data of chorionicity, aneuploid cases were DCDA. However, regarding the low number of cases, no conclusion can be made about the effect of chorionicity on the interpretation and accuracy of screening results. Yang et al. screened 432 twin pregnancies by NIPT and reported no false negative case. Based on their results, the combined false-positive rate for trisomies 21 and 18 was 0%. Yet, they reported two false positive cases (one T7 and one 47XXX) [5]. Previous studies have stated confined placental mosaicism [17], maternal mosaicism [18], malignancy [19], co-twin death [20] and uniparental disomy [5] as the sources of false positive results in NIPT. Nonetheless, we could not find any underlying reason for the false positive results in our study. Overall, NIPT is recommended in IVF-ICSI pregnancies due to their critical situations and the higher prevalence of twin pregnancies.

## Conclusion

The results of the present study verified the feasibility, sensitivity and specificity of NIPT in twin pregnancies. NIPT method, however, possesses several drawbacks which should be considered and explained during the pre-test and post-test counseling. It must be noted that placental but not fetal DNA is examined in NIPT and placenta genetic aberrations might be different from the fetus. Moreover, statistical analysis showed the failure of NIPT in about 2–6% of cases. Finally, trisomy 21 accounted for about only half of the existing chromosomal abnormalities [21]. Thus, a comprehensive explanation of these shortcomings will help in better decision-making.

## Data Availability

The datasets used and/or analyzed during the current study are available from the corresponding author on reasonable request.

## Abbreviations

DCDA: Double chorionic dichorionic diamniotic
IVF-ICSI: In vitro fertilization-intracytoplasmic sperm injection
MCDA: Monochorionic diamniotic
NIPT: Non-invasive prenatal testing
